# Comment on Saner et al. The Yin and the Yang of Hemostasis in End-Stage Liver Disease. *J. Clin. Med.* 2023, *12*, 5759

**DOI:** 10.3390/jcm13061666

**Published:** 2024-03-14

**Authors:** Bruce D. Spiess, Wim P. M. Houdijk

**Affiliations:** HemoSonics LLC, Durham, NC 27705, USA

We congratulate you on a quality review of the article on hemostasis in end-stage liver disease by Saner FH et al. [1]. Early in the article, the authors focus on the fact that many patients with end-stage liver disease have a tendency for thrombosis and hypercoagulability. This point is vital since most physicians have a cursory impression of hypocoagulability predominating in hepatic failure. The biology is far more complex, as the authors point out, and the interplay of proteins with endothelial dysfunction at the interface of vascular cells and blood is where the thrombosis occurs. The glycocalyx is the natural barrier to inflammation and coagulation. It is through antithrombin, albumin, other proteins, platelets, neutrophils, and other cell types that a nidus for thrombosis may be initiated. When the liver fails, the controlling mechanisms that buffer inflammation and coagulation (coagulation is the first part of inflammation) do not rein in the tendency for thrombosis, allowing it to mature or spread. We thank the authors for pointing out these complex relationships.

The term “standard laboratory test” (SLT) has been coined and come to mean PT, aPTT, platelet count, fibrinogen, etc. This is a misnomer and perhaps we in manuscripts should be calling these something else, such as central laboratory coagulation analysis (CLCA). Viscoelastic tests (VETs) are now the standard. There are thousands of publications regarding VETs, and one has to wonder if by calling CLCAs the standard the connotation is that VETs are somehow not acceptable or standard. How many medical publications and how widespread does the utilization of a laboratory test have to be before it is named a standard? Point-of-care (POC) VETs are called for in all patient blood management guidelines by academic societies and viscoelastic testing was found to be beneficial in reducing blood usage and improving outcomes in heart surgery [2,3,4]. Does that not call for it to be a “standard”?

The main reason, however, that we are writing to the Editor is to point out that the article has discussed only two VET technologies, neglecting a major other technology. TEG and ROTEM are mechanical technologies that arose out of the original work by H Hartert in 1947, wherein he examined clot elastic modulus with a rotating cup and piston. In 2004, sonorheometry was first published as a direct measure of clot elastic modulus utilizing medical-grade ultrasound. The Quantra^®^ system today utilizes ultrasound resonance to directly measure the physical strength of blood (shear modulus expressed in hecto-Pascals) as it changes from a liquid to a gel (semi-solid) [5,6,7,8,9]. Since its regulatory approval in Europe (2017) and the United States (2019), this technology has been marketed as the Quantra^®^ analyzer, a rapid, cartridge-based, stand-alone, point-of-care (POC) VET system. There are over 50 peer-reviewed articles regarding how the technology works and its comparison to both CLCA as well as to the pre-existing POC VET devices TEG 5000 and ROTEM *delta* and now the contemporary POC cartridge-based devices TEG 6S and ROTEM *sigma*. As you might expect, the VET platforms are apples and oranges, similar but different. Although the VET systems do describe the same biologic process with significant correlations between certain parameters, there are differences in terms of methodology and parameters.

In 2022, the Quantra QStat cartridge system was approved for marketing in liver transplantation and trauma by the US FDA. This cartridge allows for the assessment not only of clotting time (as activated through the intrinsic cascade) but it also looks for the percentage of clot lysis (CSL) with and without plasmin inhibition using tranexamic acid. The Quantra system assesses clot strength (CS) over time as a measure of physical force (shear modulus in hecto-Pascals; not an interpolated graph on a 0–100 mm arbitrary scale) and further breaks that analysis into contributions by fibrinogen (FCS) and platelet activity (PCS).

The literature regarding Quantra sonorheometry, which it appears Saner et al. were unaware of, should be made known to your readers. Like TEG and ROTEM, Quantra sonorheometry is a commercially available option for the care of hepatic patients. Recent work with Quantra shows it being utilized during liver transplantation [10]. Data emerging from the cardiac surgery literature show that the Quantra system is the fastest point-of-care VET system on the market [11]. Furthermore, when it has been systemically instituted, it has improved patient outcomes as well as saved blood utilization and reduced wastage, and thereby has been cost-effective [12,13].

The review article by Saner et al. points out that VET can not only be utilized for the assessment of bleeding risks but also for the tendency to thrombose. Since hepatic failure thrombosis is related to complex endothelial glycocalyx dysfunction, systemic VET might show tendencies for hypercoagulability but the events themselves occur at sites of vascular injury to which VET is insensitive.

Future research with the various POC-VET tests will be required to understand how these tests may help in the complex biology of end-stage liver disease.
